# Calculation of Apparent Diffusion Coefficients in Prostate Cancer Using Deep Learning Algorithms: A Pilot Study

**DOI:** 10.3389/fonc.2021.697721

**Published:** 2021-09-09

**Authors:** Lei Hu, Da Wei Zhou, Cai Xia Fu, Thomas Benkert, Yun Feng Xiao, Li Ming Wei, Jun Gong Zhao

**Affiliations:** ^1^Department of Diagnostic and Interventional Radiology, Shanghai Jiao Tong University Affiliated Sixth People’s Hospital, Shanghai, China; ^2^State Key Laboratory of Integrated Services Networks, School of Telecommunications Engineering, Xidian University, Xi’an, China; ^3^Magnetic Resonance (MR) Application Development, Siemens Shenzhen Magnetic Resonance Ltd., Shenzhen, China; ^4^MR Application Predevelopment, Siemens Healthcare GmbH, Erlangen, Germany

**Keywords:** apparent diffusion coefficient, diffusion magnetic resonance imaging, deep learning, prostatic neoplasms, supervised machine learning

## Abstract

**Background:**

Apparent diffusion coefficients (ADCs) obtained with diffusion-weighted imaging (DWI) are highly valuable for the detection and staging of prostate cancer and for assessing the response to treatment. However, DWI suffers from significant anatomic distortions and susceptibility artifacts, resulting in reduced accuracy and reproducibility of the ADC calculations. The current methods for improving the DWI quality are heavily dependent on software, hardware, and additional scan time. Therefore, their clinical application is limited. An accelerated ADC generation method that maintains calculation accuracy and repeatability without heavy dependence on magnetic resonance imaging scanners is of great clinical value.

**Objectives:**

We aimed to establish and evaluate a supervised learning framework for synthesizing ADC images using generative adversarial networks.

**Methods:**

This prospective study included 200 patients with suspected prostate cancer (training set: 150 patients; test set #1: 50 patients) and 10 healthy volunteers (test set #2) who underwent both full field-of-view (FOV) diffusion-weighted imaging (f-DWI) and zoomed-FOV DWI (z-DWI) with *b*-values of 50, 1,000, and 1,500 s/mm^2^. ADC values based on f-DWI and z-DWI (f-ADC and z-ADC) were calculated. Herein we propose an ADC synthesis method based on generative adversarial networks that uses f-DWI with a single *b*-value to generate synthesized ADC (s-ADC) values using z-ADC as a reference. The image quality of the s-ADC sets was evaluated using the peak signal-to-noise ratio (PSNR), root mean squared error (RMSE), structural similarity (SSIM), and feature similarity (FSIM). The distortions of each ADC set were evaluated using the T2-weighted image reference. The calculation reproducibility of the different ADC sets was compared using the intraclass correlation coefficient. The tumor detection and classification abilities of each ADC set were evaluated using a receiver operating characteristic curve analysis and a Spearman correlation coefficient.

**Results:**

The s-ADC_b1000_ had a significantly lower RMSE score and higher PSNR, SSIM, and FSIM scores than the s-ADC_b50_ and s-ADC_b1500_ (all *P* < 0.001). Both z-ADC and s-ADC_b1000_ had less distortion and better quantitative ADC value reproducibility for all the evaluated tissues, and they demonstrated better tumor detection and classification performance than f-ADC.

**Conclusion:**

The deep learning algorithm might be a feasible method for generating ADC maps, as an alternative to z-ADC maps, without depending on hardware systems and additional scan time requirements.

## Introduction

Diffusion-weighted imaging (DWI) currently constitutes an integral part of multiparametric magnetic resonance imaging (MRI) examinations of the prostate. Apparent diffusion coefficients (ADCs) obtained with DWI are highly valuable for detecting and staging prostate cancer, evaluating cancer aggressiveness (1, 2), guiding targeted biopsies, and assessing the response to treatment (3–10). Clinically, the accuracy of the ADC measurement depends on the quality of the DWI image.

Single-shot echo-planar imaging (SS-EPI)-based sequences are preferred for DWI because of its ability to acquire the images rapidly and the robustness of the technique against motion artifacts. However, because of its high sensitivity to chemical shifts and magnetic susceptibilities (11), conventional SS-EPI DWI suffers from significant anatomic distortions (12) and susceptibility artifacts, resulting in reduced ADC calculation accuracy and reproducibility (12–14). Another limitation is the low signal-to-noise ratios observed during DWI, which result in noise-induced signal intensity biases (15, 16) and inaccurate ADC maps. These drawbacks may lead to an error in judgment regarding the condition of a patient and a potential misdiagnosis of malignant lesions or over-treatment of benign lesions. Zoomed field-of-view (FOV) DWI (z-DWI) is an appealing attempt to address these limitations. This method reduces the scanning time as well as artifacts, distortions, and blurring of images, and it also has improved spatial resolution (17, 18). Additionally, z-DWI can effectively improve the ADC map accuracy (17, 18); however, the technique depends on radio frequency design and software platforms (17–19), which can make it unaffordable for many small- and medium-sized hospitals and their patients. Moreover, a reduced FOV may prevent the visualization of lymph nodes (3). Therefore, the clinical application of z-DWI is limited. A method that can consistently generate high-quality ADC images with reduced equipment costs will be of more benefit to patients in clinical practice.

Recently, the advent of generative adversarial networks (GANs) (20) has shown promise for optimizing medical image quality without relying on software and equipment conditions (21). As a generative model, the objective of a GAN is to learn the underlying training data distributions to generate realistic images that are indistinguishable from the input datasets (21). With their ability to mimic data distributions, GANs have been used to translate low-quality images into high-quality counterparts. Previous studies have successfully used GANs to improve computed tomography (CT) or MRI quality in terms of de-noising (22), increased resolution (23), artifact reduction (24), and motion correction (25). Inspired by these image optimization solutions, we hypothesized that deep learning algorithms based on GANs might be promising for generating ADC maps with good image quality and improved ADC calculation accuracy. The purpose of this study was to establish and evaluate a supervised learning framework based on a GAN to synthesize realistic zoomed FOV ADC images using conventional full FOV SS-EPI DWI images with a single *b*-value.

## Materials and Methods

### Patients and Healthy Volunteers

**Figure 1:**
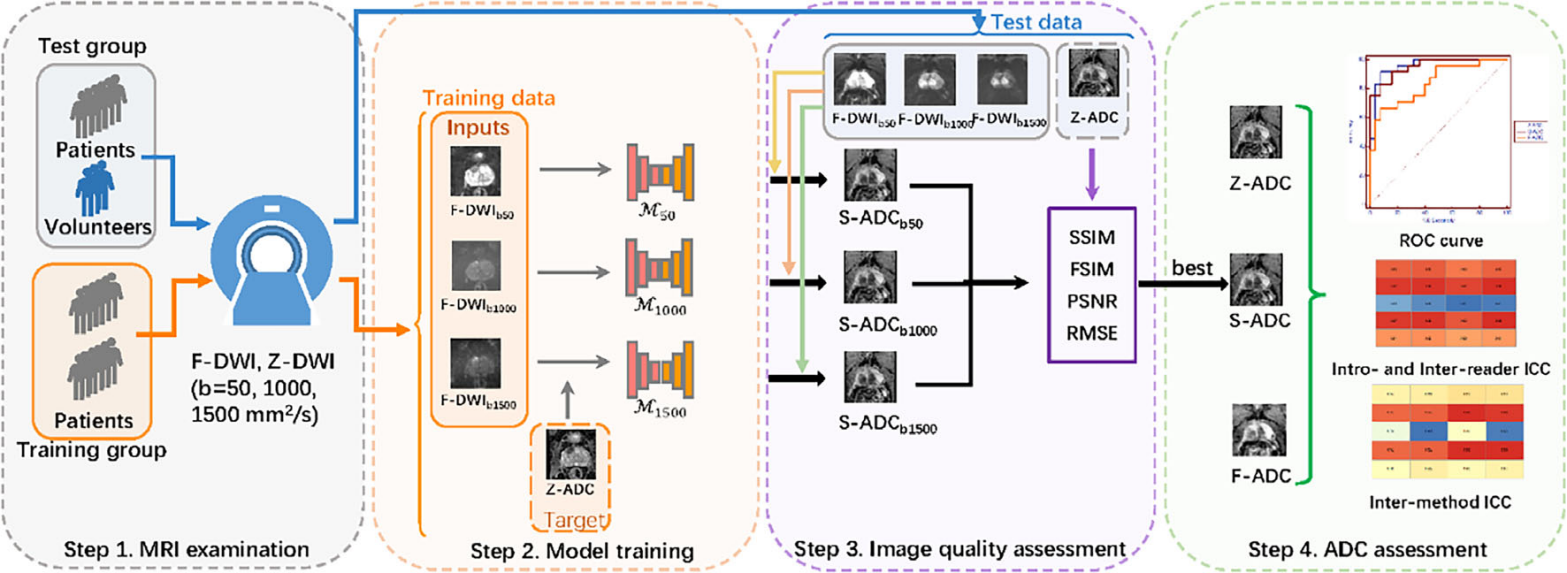
Overall study flow diagram. Step 1: All the patients and healthy volunteers underwent multiparametric magnetic resonance imaging examinations of the prostate, including full field-of-view (FOV) diffusion-weighted imaging (f-DWI) and zoomed FOV diffusion-weighted imaging with *b*-values of 50, 1,000, and 1,500 s/mm^2^. Step 2: The models that used full f-DWI with different *b*-values (f-DWI_b50_, f-DWI_b1000_, and f-DWI_b1500_) to synthesize the apparent diffusion coefficient (s-ADC) maps (s-ADC_b50_, s-ADC_b1000_, and s-ADC_b1500_) were trained. Step 3: The image quality of s-ADC_b50_, s-ADC_b1000_, and s-ADC_b1500_ were evaluated using the peak signal-to-noise ratio, root mean square error, structural similarity, and feature similarity. Step 4: An ADC assessment was performed to determine reproducibility, tumor detection, and classification.

This prospective study was approved by the local ethics committee, and informed consent was obtained from each participant. All the procedures involving human participants were performed in accordance with the 1964 Helsinki Declaration and its later amendments. A total of 200 consecutive patients underwent preoperative MRI examinations and subsequent MRI fusion ultrasound-guided biopsies for suspected prostate cancer (PCa) between December 2018 and May 2020. The inclusion criteria were as follows: patients with (1) at least one prostate lesion visible on DWI and ADC maps and (2) complete clinical information and pathologic examination information, including biopsy reports. Ten healthy volunteers were also recruited for the study. The study included four steps: (1) MRI examinations, (2) model training, (3) image quality assessments, and (4) ADC assessments (**Figure 1**).

### MRI Examinations and Datasets

All the patients and volunteers underwent multiparametric MRI examinations of the prostate using a 3T MRI scanner (MAGNETOM Skyra, Siemens Healthcare, Erlangen, Germany) equipped with a phased-array 18-channel body coil and an integrated 32-channel spine coil. Both a transversal single-shot full FOV-EPI DWI (f-DWI) and a prototypic non-parallel transmission zoomed EPI DWI (z-DWI) with *b*-values of 50, 1,000, and 1,500 s/mm^2^ were performed with the ADC reconstruction maps (f-ADC and z-ADC) using a standard mono-exponential with all the acquired *b*-values (14). Axial T2-weighted images were obtained from all the participants, and the total examination time was approximately 7 min and 40 s. The detailed scan parameters are shown in **Table 1**.

**Table 1:** The magnetic resonance imaging sequence parameters.

Parameter	T2-weighted imaging	F-DWI	Z-DWI
Field-of-view, FOV (mm^2^)	200 × 200	380 × 280	190 × 106
Imaging matrix	320 × 320	132 × 178	112 × 200
Thickness (mm)	3.5	3	3
Distance fact	0	10%	10%
B-value (s/mm^2^)	n.a.	50, 1,000, 1500	50, 1,000, 1500
Echo time (ms)	101	73	76
Time to repeat (ms)	6,000	4,200	3,800
Bandwidth (Hz/pixel)	200	1,872	1,612
Scan time (min)	2:08	3:05	2:27

f-ADC, mean apparent diffusion coefficient (ADC) map derived from full FOV diffusion-weighted imaging with all available b-values (b = 50, 1,000, and 1,500 mm^2^/s); z-ADC, ADC map derived from zoomed FOV diffusion-weighted imaging with all available b-values (b = 50, 1,000, and 1,500 mm^2^/s), n.a., no available.

Patient images were randomly divided into two groups (training set: 150 patients, test set #1: 50 patients). The training set was used to build the framework and train different models to synthesize the ADC maps (s-ADCs). Test set #1 was used to test the reproducibility of the s-ADC prostate lesion measurements, along with tumor detection. The images of the healthy volunteers were regarded as test set #2, which was used to test the reproducibility and consistency of the normal prostate tissue s-ADC calculations, including the peripheral zone (PZ) and the transitional zone (TZ).

### Data Pre-Processing

Before the model training could occur, image selection, cropping, and registration were performed on f-DWI with *b*-values of 50, 1,000, and 1,500 s/mm^2^ and the z-ADC images. The first and last slices that did not cover the prostate were removed manually. The images with severe distortion and artifacts were also removed. Ultimately, there were between five and 20 DWI images selected for each person. Finally, there were 2,250 images from each set for the 150 patients in the training set, 750 images from each set for the 50 patients in test set #1, and 145 images from each set for the 10 healthy volunteers in test set #2.

Due to hardware limitations of the graphics cards and the CPU memory, we used only axial slices of the cropped data to train the two-dimensional generation models. The f-DWI data had an original voxel size of 2.13 × 2.13 × 3.3 mm^3^ and a matrix size of 178 ×132, whereas the z-ADC data had a voxel size of 0.95 × 0.95 × 3.3 mm^3^ and a matrix size of 112 × 200. The f-DWI data were first resampled to a voxel size of 0.95 × 0.95 × 3.3 mm^3^ with a matrix size of 360 × 267, and both modalities were cropped at the center to extract the relevant prostate region. The f-DWI data were then aligned to the z-ADC data using the affine transformation implemented by the Advanced Normalization Tools (https://github.com/ANTsX/ANTs). To facilitate the model training, all the two-dimensional axial slices were scaled to a unified resolution of 224 × 224 pixels.

To select a suitable *b*-value for ADC synthesis, we first used 2,250 paired f-DWI images with *b*-values of 50 s/mm^2^ and the ground truth z-ADC maps from the training set as inputs and references, respectively, to train our framework-based model M_50_ to synthesize ADC maps (s-ADC_b50_). Similarly, the M_1000_ and M_1500_ models based on the f-DWI images with *b*-values of 1,000 and 1,500 s/mm^2^ were trained to synthesize ADC maps (s-ADC_b1000_ and s-ADC_b1500_).

### Model Training

**Figure 2:**
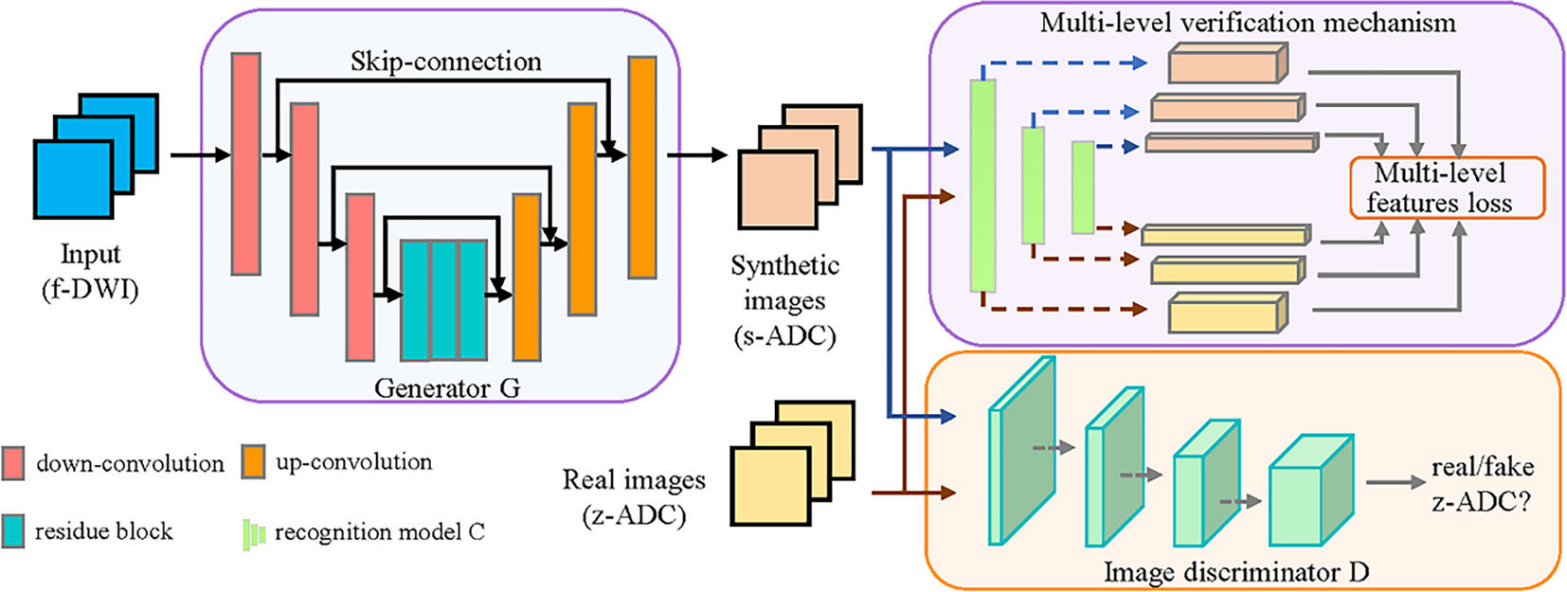
Illustration of our framework. The proposed framework consists of a generator (*G*), which was constructed using a deep convolution network with skip connections, and an image discriminator (*D*) constructed using a full convolution network. The *G* transforms the f-DWI into a synthesized apparent diffusion coefficient (s-ADC) using zoomed field-of-view diffusion-weighted imaging (z-ADC) as a reference. The *D* takes either s-ADC or z-ADC as the input and determines whether the input is a real z-ADC. In addition, to promote *G* in an effort to retain better features for diagnosis, we introduced a multi-level verification mechanism, including a pre-trained recognition model (*C*), to extract the multi-level features from the s-ADC and the z-ADC.

We have proposed a GAN-based framework to generate realistic z-ADC maps from f-DWI maps (**Figure 2**).

The generator *G* translates the input image (f-DWI) *X* into the synthesized ADC (s-ADC) *S* with a quality similar to the ground truth z-ADC, which is used as reference *Y*. The discriminator *D* takes either the *S* or the *Y* as input and determines whether the input is a real z-ADC map. Specifically, we used a deep residual network structure with skip connections to construct the generator *G* and a full convolution network to construct the discriminator *D*. The adversarial loss of the discriminator *D* is formulated as follows:

$${ℒ}_{D}=E_{Y∼P_{Y}}[{\left(D(Y)-1\right)}^{2}]+E_{S∼P_{S}}[{\left(D(S)\right)}^{2}]$$

The adversarial loss of the generator *G* is formulated as follows:

$${ℒ}_{G}^{adv}=E_{S∼P_{S}}[{\left(D(S)-1\right)}^{2}]$$

Considering that the standard GAN might not adequately preserve the tumors/lesions during image-to-image translation (26), we introduced a multi-level verification (MLV) mechanism, including a pre-trained recognition model *C*. This mechanism promotes the generator *G* to better retain the features, which helps in the diagnosis. Using *C*, the proposed MLV mechanism provides more details about the tumor/lesion features when they are extracted from the input images. *G* represents the tumor/lesion texture, making it better and more robust against changes in appearance and geometric transformations (27).

We first obtained a recognition model *C*, which was pre-trained on a VGG-19-based network using the processed images from the patients and healthy volunteers with a benign or malignant label (28). Subsequently, the multiple layers of model *C* extracted the multi-level features from the fake synthetic ADC map *S* and the ground truth ADC map. The sum of the mean square errors of the features in each level layer was used as the multi-level feature loss to supervise the generator *G*.

Inspired by the current work (29) and considering the use of multi-level features, we selected the features in the 0, 1st, 3rd, and 5th level layers. The loss of the multi-level verification mechanism is formulated as follows:

$${ℒ}_{G}^{mlv}=\Sigma_{i=0,1,3,5}\theta_{i}\cdot {\left||C^{i}(S)-C^{i}(Y)|\right|}_{2}^{2}$$

where *θ_i_* ∈ (0, 1) denotes the weight parameter for the loss $\left({ℒ}_{mlf}^{i}\right)$ at different levels, and it is optimized in each epoch to cause a faster decrease in the loss of the larger items. The *θ_i_* in the *j - th* epoch can be computed as follows:

$$\theta_{i}^{j}=\Sigma_{n=0,1,3,5}\frac{{\left||C_{i}^{j-1}(S)-C_{i}^{j-1}(Y)|\right|}_{2}^{2}}{{\left||C_{n}^{j-1}(S)-C_{n}^{j-1}(Y)|\right|}_{2}^{2}}$$

where $C_{i}^{j-1}(\cdot )$ denotes the feature of the *i - th* layer in the *(j-*1*)-th* epoch, and $C_{n}^{j-1}(\cdot )$ indicates the feature of the *n - th* layer in the *(j-*1*)-th* epoch. We initialized *θ_i_* to 1/4. The objective function of generator *G* is formulated as follows:

$${ℒ}_{G}={ℒ}_{G}^{adv}+\lambda_{1}{ℒ}_{G}^{mlv}$$

with γ_1_ set to 10^-1^.

### Experimental Settings

The generator consists of three convolution layers, followed by five residual blocks and three deconvolution layers. Each convolution or deconvolution layer is followed by an instance-normalization layer and a ReLu activation layer. The discriminator consists of five convolution layers. The learning rate was set to 0.001 for both the generator and the discriminator. The batch size was set to 5, and the epoch was set to 50. The details of the generator and discriminator can be found at https://github.com/huxiaolie/ADC_generation. All the algorithms were implemented using Python 3.6 (https://www.python.org/downloads/release/python-362/) and Pytorch 1.6.0 (https://pytorch.org/get-started/previous-versions/) on an Ubuntu 16.04 system with an NVIDIA TITAN XP GPU.

### Image Quality Assessment

The s-ADC sets were synthesized using each model with inputs from the f -DWI images with *b*-values of 50, 1,000, and 1,500 s/mm^2^ for test set #1 (50 patients) and test set #2 (10 healthy volunteers), and they were compared using peak signal-to-noise ratios (PSNRs), root mean square errors (RMSEs), structural similarities (SSIMs), and feature similarities (FSIMs) (30).

A radiologist with 6 years of experience with prostate MRIs measured the anterior–posterior (AP) and left–right (LR) diameters of each prostate on the ADC set on the slice on which the prostate showed the greatest cross-sectional area. The differences in the measured AP and LR diameters of the prostate relative to the T2-weighted image (T2WI) were computed for f-ADC, z-ADC, and s-ADC, with the best performance from the above-mentioned quantitative evaluation.

### ADC Measurement Assessment

For the patient study, two radiologists with 5 and 10 years of experience with prostate MRIs and who were unaware of the clinical, surgical, and histologic findings independently drew a circular region of interest (ROI) with an area of approximately 0.5–0.8 cm^2^ in the center of the lesion, excluding its edges. For the healthy volunteer study, the readers drew circular ROIs with an area of approximately 0.5 cm^2^ in the peripheral and transitional zones on the ADC maps using axial T2-weighted images as the anatomical reference. The mean ADC values for each ROI were recorded.

The ADC sets of all the patients and healthy volunteers were measured twice using Image J (NIH Image, Bethesda, MD) in a different order, with an interval of 2 weeks. The first measurement given by the two readers showed the consistency of the ADC measurements for each ADC set. The second measurement showed the repeatability of the ADC values for each ADC set.

### Tumor Detection Assessment

The s-ADC set with the best image quality and ADC measurement assessment among the three s-ADC sets was selected for tumor detection assessments. The selected s-ADC was compared with the f-ADC and z-ADC in terms of the ability to differentiate benign from malignant lesions. The correlation between the ADC values in the different ADC sets and tumor grades was also evaluated.

### Statistical Analyses

Analyses of the baseline characteristics between the training group and the test group were conducted. An independent *t*-test was used to assess normally distributed continuous variables. The Mann–Whitney *U*-test was used to assess non-normally distributed continuous variables.

To assess differences in the image quality metrics (PSNR, RMSE, SSIM, and FSIM) between any two s-ADC sets, a paired Student’s *t*-test was applied. The intraclass correlation coefficient (ICC) was used to assess the inter-and intra-reader repeatability of the ADC measurements for each tissue (malignant lesion, benign lesion, peripheral zone, and transitional zone) in each ADC set (f-ADC, z-ADC, and s-ADC). The ICC was also used to evaluate the inter-method reliability of the ADC values for each tissue between the synthesized image (s-ADC) and the reference image (z-ADC). A receiver operating characteristic (ROC) curve analysis was performed to assess the ability to discriminate between benign and malignant prostate lesions based on the ADC values. The differences in the area under the curve (AUC) values were tested using DeLong tests. The statistical analyses were performed using MedCalc software. Two-tailed tests were used to calculate all the *P*-values. Statistical significance was set at *P <*0.05.

## Results

### Demographic Characteristics

The patient characteristics are summarized in **Table 2**. There were no significant differences in the mean ages between the patients with and without PCa (*P* = 0.557). The mean prostate-specific antigen (PSA) level was significantly higher in patients with PCa compared to those without PCa (*P* < 0.001).

**Table 2:** The clinical characteristics of the patient cohort.

Characteristics	Patients without cancer (*n* = 106)	Patients with cancer (*n* = 94)	*P*-value
Mean age (y) [range]	70 (52–87)	71 (48–88)	0.675
total PSA (ng/ml)	11.079 ± 9.013	57.002 ± 125.88	<0.001
Position, no.			
Peripheral zone	44	63	<0.001
Transitional zone	62	31	
Gleason score (*n*, %)			
6	——	8	
7	——	46	
8	——	24	
9	——	16	

The data are mean ± standard deviation, unless otherwise indicated.

PSA, prostate-specific antigen.

There were no significant differences in mean ages and mean PSA between the training set and test set #1 (mean ages: 68 ± 10 *vs*. 68 ± 12 years, *P* = 0.974; PSA: 29.872 ± 69.461 *vs*. 39.296 ± 92.604, *P* = 0.154). The mean age of test set #2 (healthy volunteers, 24 ± 3 years) is significantly lower than that of the training set and test set #1 (*P* < 0.001).

### Image Quality Assessment

**Figure 3:**
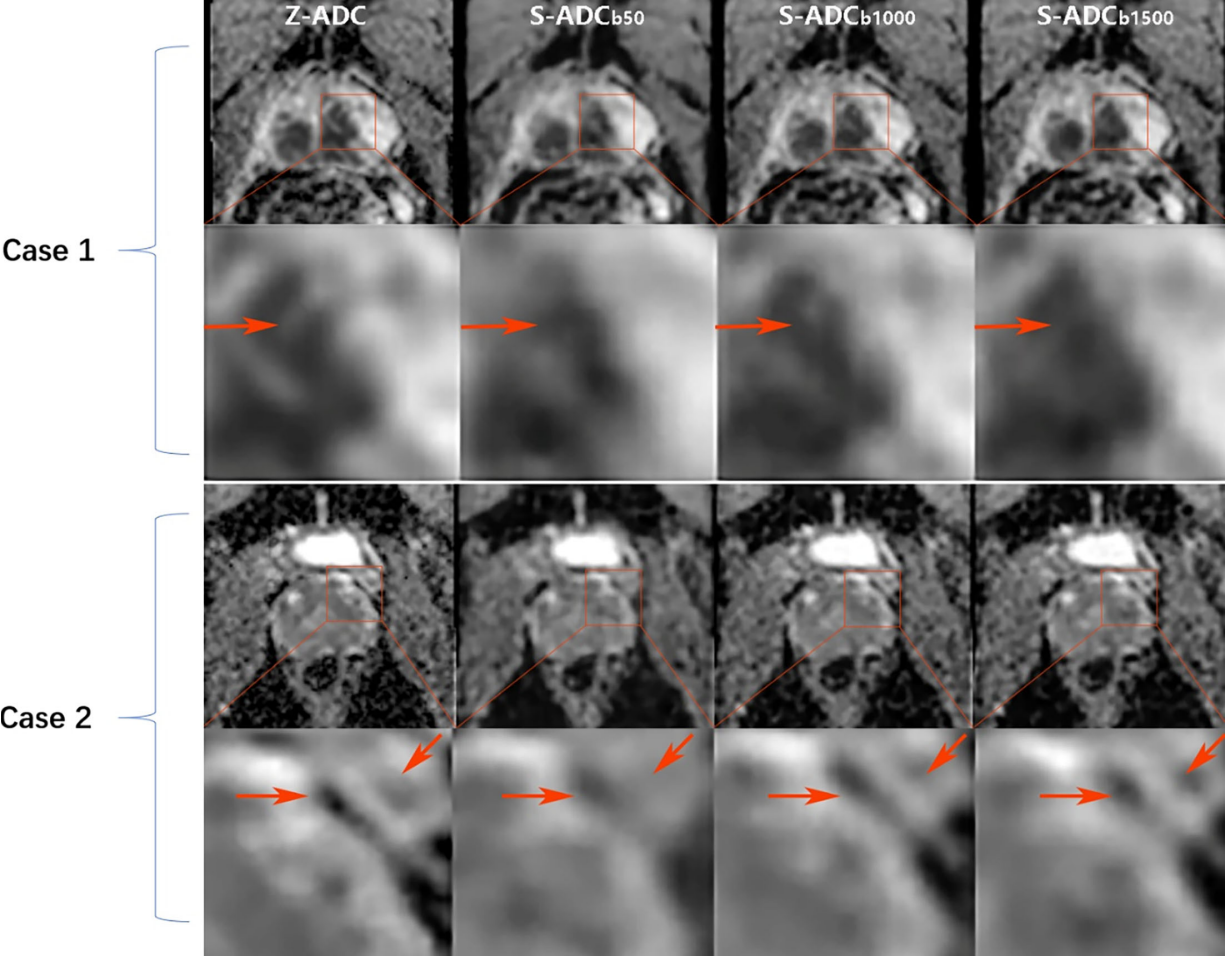
Comparison of the synthesized apparent diffusion coefficient (s-ADC) maps. Case 1: An 82-year-old man with prostate cancer from test set 1 who had an initial prostate-specific antigen level of 13.04 ng/ml. Case 2: A 27-year-old healthy man from test set 2. For these two cases, both the s-ADC_b1000_ and s-ADC_b1500_ performed well in displaying the prostate, pelvic floor muscles, pubic symphysis, and the entire cancer lesion. However, the s-ADC_b50_ images of these structures are fuzzy. According to the local enlargement of the images (the images in the second and fourth lines), the s-ADC_b1000_ is more similar to the z-ADC than to the s-ADC_b1500_, and it retains more details of the z-ADC (shown as red arrows).

Visual comparisons of the s-ADC values generated with different *b*-value inputs are shown in **Figure 3**. We observed that the s-ADC_b50_ displayed blurred images of the prostate, bladder, rectum, pelvic floor muscles, and pubic symphysis in both the patients and the volunteers. Compared with s-ADC_b50_, s-ADC_b1000_ and s-ADC_b1500_ could delineate normal tissues and lesions more clearly and sharply, which was in line with the ground truth. According to the magnified images of the local tissue structures, s-ADC_b1000_ provided more details than s-ADC_b1500_ with reference to z-ADC.

**Figure 4:**
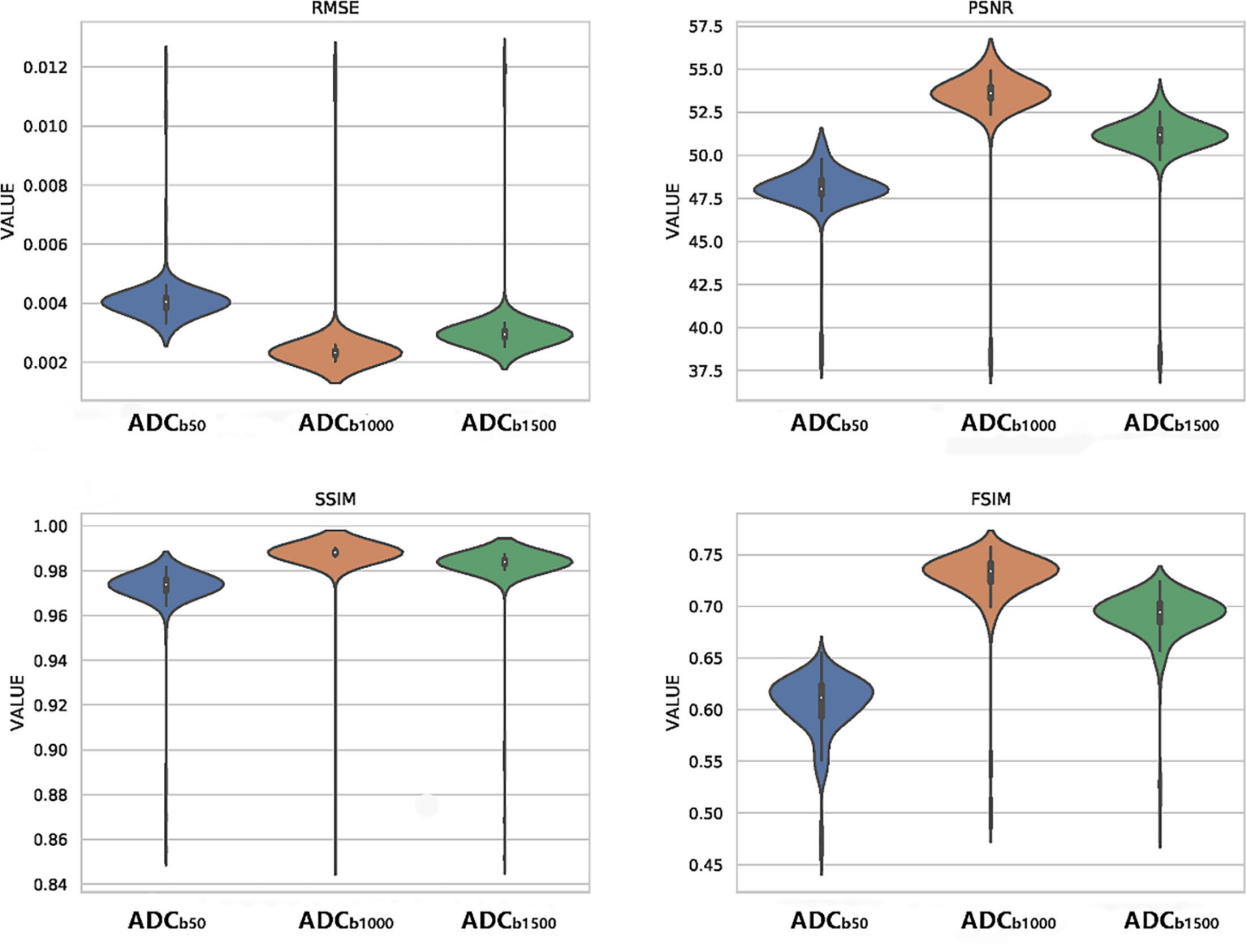
Violin plots of the quantitative metric distributions of the s-ADC sets.

As shown in the violin plots (**Figure 4**), s-ADC_b1000_ performed better than the other two s-ADC sets in terms of the distribution, median, and inter-quartile ranges of the RMSE, SSIM, FSIM, and PSNR scores. The mean RMSE scores of s-ADC_b50_, s-ADC_b1000_, and s-ADC_b1500_ were 4.1 × 10^-3^, 2.5 × 10^-3^, and 3.1 × 10^-3^, respectively. The mean PSNR scores of s-ADC_b50_, s-ADC_b1000_, and s-ADC_b1500_ were 48.0, 53.4, and 51.0, respectively. The mean SSIM scores of s-ADC_b50_, s-ADC_b1000_, and s-ADC_b1500_ were 0.972, 0.986, and 0.982, respectively. The mean FSIM scores of s-ADC_b50_. s-ADC_b1000_, and s-ADC_b1500_ were 0.604, 0.728, and 0.690, respectively. s-ADC_b1000_ had a significantly lower RMSE score and higher PSNR, SSIM, and FSIM scores than s-ADC_b50_ and s-ADC_b1500_ (all *P* < 0.05).

To evaluate the distortion in the ADC maps, the differences in the AP and LR diameters of the prostate relative to T2WI were both significantly lower for s-ADC (AP, 2.734; LR, 3.204) and z-ADC (AP, 2.755; LR, 3.073) than for f-DWI (AP, 5.916; LR, 5.053) (all *P* < 0.001).

### ADC Measurement Assessment

The measurements of all the ADC sets (f-ADC, z-ADC, s-ADC_b50_, ADC_b1000_, and s-ADC_b1500_) on various tissues from both readers are presented in **Table 3**. For all the ADC sets, the ADC values of the TZ are significantly lower than those of the PZ, while the ADC values of the malignant lesions are significantly lower than those of the benign lesions (all *P* < 0.05).

**Table 3:** The mean apparent diffusion coefficient (ADC) values (×10^-3^ mm^2^/s) of the different ADC sets.

Parameter	ADC value (×10^-3^ mm^2^/s)			
	Peripheral zone (*n* = 10)	Transitional zone (*n* = 10)	Benign lesions (*n* = 26)	Malignant lesions (*n* = 24)
Reader 1				
f-ADC	1.90 ± 0.11	1.41 ± 0.13	1.40 ± 0.28	1.06 ± 0.25
z-ADC	1.43 ± 0.17	1.20 ± 0.16	0.98 ± 0.18	0.61 ± 0.11
s-ADC_b50_	1.43 ± 0.25	1.20 ± 0.18	1.09 ± 0.23	0.68 ± 0.13
s-ADC_b1000_	1.43 ± 0.16	1.20 ± 0.16	0.99 ± 0.18	0.61 ± 0.17
s-ADC_b1500_	1.46 ± 0.18	1.26 ± 0.16	1.01 ± 0.17	0.67 ± 0.18
Reader 2				
f-ADC	1.94 ± 0.14	1.39 ± 0.19	1.42 ± 0.29	1.06 ± 0.25
z-ADC	1.49 ± 0.16	1.22 ± 0.14	0.98 ± 0.18	0.61 ± 0.11
s-ADC_b50_	1.44 ± 0.13	1.18 ± 0.14	1.02 ± 0.24	0.69 ± 0.13
s-ADC_b1000_	1.48 ± 0.21	1.18 ± 0.13	0.99 ± 0.16	0.61 ± 0.15
s-ADC_b1500_	1.45 ± 0.12	1.18 ± 0.09	1.00 ± 0.16	0.70 ± 0.10

The ADC values of the lesions were calculated using images from the patients in test set 1. The ADC values of the normal prostate tissues in the peripheral and transitional zones were calculated using images from the healthy volunteers in test set 2.

f-ADC, ADC map derived from full field-of-view (FOV) diffusion-weighted imaging (f-DWI) with all available b-values (b =50, 1,000, and 1,500 s/mm^2^); z-ADC, ADC map derived from the zoomed FOV diffusion-weighted imaging and all available b-values (b = 50, 1,000, and 1,500 s/mm^2^); s-ADC_b50_, ADC map synthesized using our proposed deep learning framework with input from the f-DWI (b = s/mm^2^); s-ADC_b1000_, ADC map synthesized using our proposed deep learning framework with input from the f-DWI (b =1,000 s/mm^2^); s-ADC_b1500_, ADC map synthesized using our proposed deep learning framework with input from the f-DWI (b =1,500 s/mm^2^).

**Figure 5:**
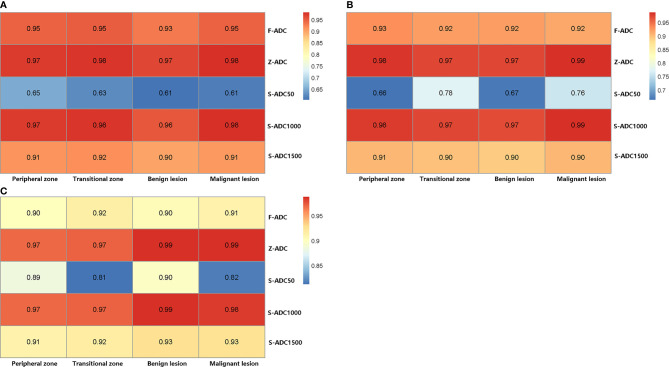
The reproducibility of the apparent diffusion coefficient (ADC) measurements as evaluated by the intraclass correlation coefficient. **(A)** The ADC measurement repeatability of reader 1 and **(B)** reader 2, and **(C)** the consistency of the ADC measurements between readers 1 and 2.

**Figure 5** presents the results of the intra-reader reproducibility (**Figures 5A, B**) and inter-reader consistency (**Figure 5C**) analyses for each ADC set calculation. Both readers reported that the reproducibility of the ADC measurements for f-ADC, z-ADC, s-ADCb1000, and s-ADCb1500 was excellent for all the tissues, while the reliability of the ADC measurements for s-ADC_b50_ was good. The inter-reader consistency of all the ADC set measurements was excellent for all the tissues. **Table 4** shows the consistency of the ADC values between the z-ADC and s-ADC sets. The consistency of the ADC values in the transitional zone between z-DWI and s-DWI_b50_ was good, and the consistency of the ADC values between z-ADC and s-ADC_b50_ for the remaining tissues was excellent. For the s-ADC_b1000_ and s-ADC_b1500_ values, the consistency of the ADC values for z-ADC for all the tissues was excellent.

**Table 4:** Comparison between the inter-method intraclass correlation coefficients from the z-DWI and s-DWI sets.

Parameter	Inter-method intraclass correlation coefficient		
	s-apparent diffusion coefficient (ADC)_b50_ *vs*. z-ADC	s-ADC_b1000_ *vs*. z-ADC	s-ADC_b1500_ *vs*. z-ADC
Reader 1			
Peripheral zone (*n* = 10)	0.87 (0.76–0.98)	0.99 (0.99–1.00)	0.99 (0.94–1.00)
Transitional zone (*n* = 10)	0.78 (0.58–0.98)	0.98 (0.87–1.00)	0.95 (0.73–0.99)
Benign lesion (*n* = 50)	0.86 (0.74–0.99)	0.98 (0.94–1.00)	0.98 (0.95–0.99)
Malignant lesion (*n* = 50)	0.89 (0.76–0.95)	0.90 (0.88–0.98)	0.88 (0.74–0.95)
Reader 2			
Peripheral zone (*n* = 10)	0.81 (0.61–0.99)	0.99 (0.97–1.00)	0.98 (0.88–1.00)
Transitional zone (*n* = 10)	0.78 (0.58–0.98)	0.99 (0.93–1.00)	0.97 (0.76–1.00)
Benign lesion (*n* = 50)	0.86 (0.73–0.99)	0.98 (0.95–0.99)	0.97 (0.93–0.99)
Malignant lesion (*n* = 50)	0.82 (0.70–0.94)	0.88 (0.72–0.95)	0.88 (0.72–0.95)

z-ADC, ADC map derived from zoomed field-of view (FOV) diffusion-weighted imaging and all the available b-values (b = 50, 1,000, and 1,500 s/mm^2^); s-ADC_b50_, ADC map synthesized using our proposed deep learning framework with input from full FOV diffusion-weighted imaging (f-DWI) (b = 50 s/mm^2^); s-ADC_b1000_, ADC map synthesized using our proposed deep learning framework with input from f-DWI (b = 1,000 s/mm^2^); s-ADC_b1500_, ADC map synthesized using our proposed deep learning framework with input from f-DWI (b = 1500 s/mm^2^).

### Tumor Detection Assessment

**Figure 6:**
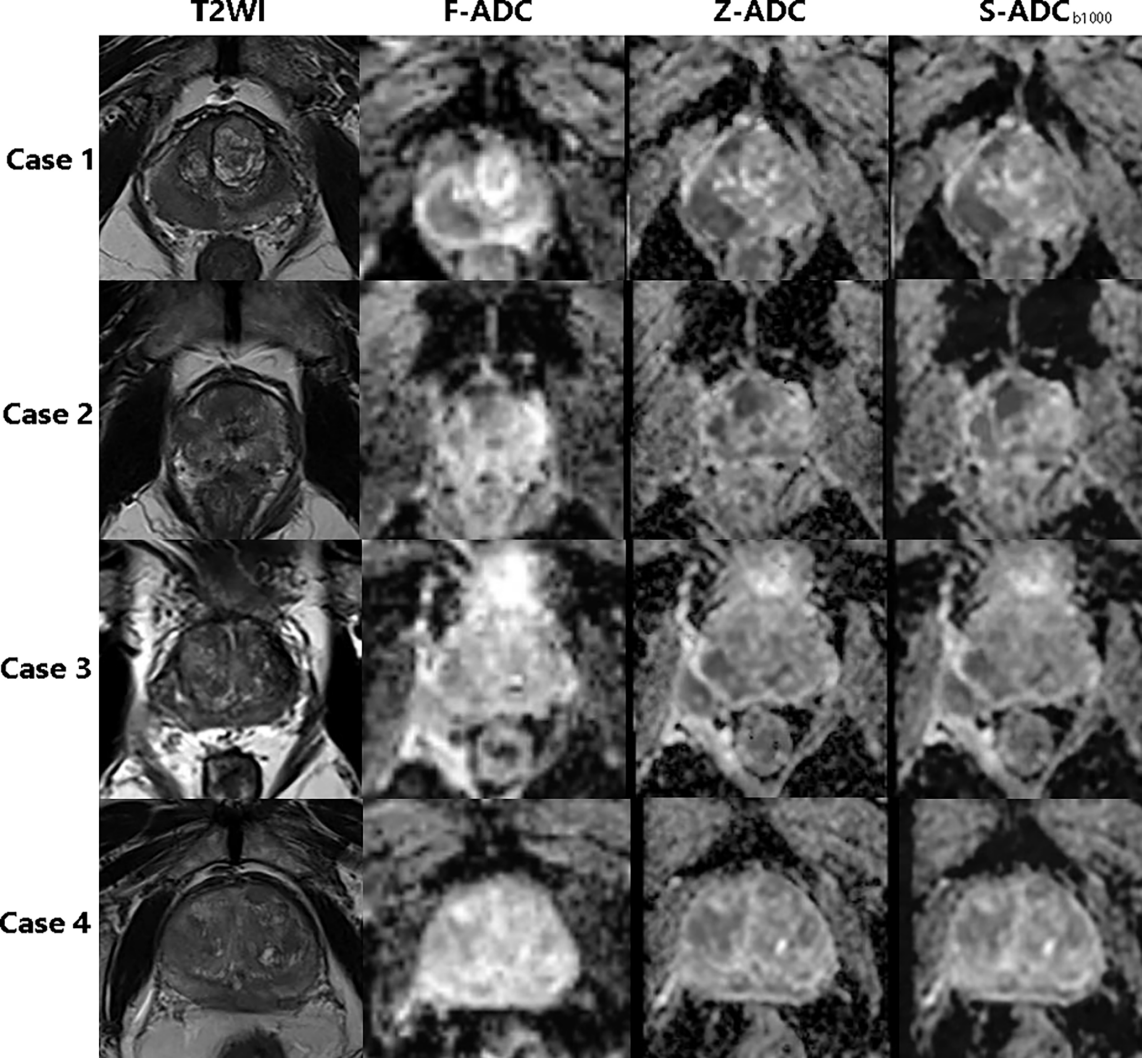
The T2-weighted image, f-ADC, z-ADC, and s-ADC_b1000_ of four different patients. Case 1: An 85-year-old man with prostate cancer in the right peripheral zone and an initial prostate-specific antigen level of 0.157 ng/ml. Case 2: An 85-year-old man with prostate cancer in the central zone and an initial prostate-specific antigen level of 21.44 ng/ml. Case 3: A 67-year-old man with an inflammatory nodule in the right peripheral zone and an initial prostate-specific antigen level of 14.37 ng/ml. Case 4: A 77-year-old man with prostate cancer in the central zone and an initial prostate-specific antigen level of 56.62 ng/ml.

**Figure 7:**
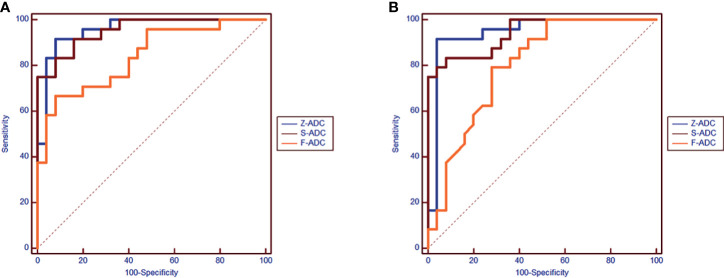
The receiver operating characteristic comparison of the diagnostic accuracy of the f-ADC, z-ADC, and s-ADCb1000 sets (**A**: reader 1, **B**: reader 2).

Among the three s-ADC sets, s-ADC_b1000_ performed the best in the image quality assessment and ADC evaluation. Therefore, it was selected for further comparisons with f-ADC and z-ADC in terms of tumor detection and classification (**Figure 6**). The ADC values for patients with malignant lesions and those with benign lesions measured by the two readers were used to compute the ROC curves (**Figure 7**). The comparisons of AUCs for both readers based on the f-ADC, z-ADC, and s-ADC sets are summarized in **Table 5**. Both the z-ADC and s-ADC sets showed significantly better predictive capabilities than the f-ADC set (*P* ≤ 0.027). The differences in AUCs between s-ADC and z-ADC were not statistically significant (reader 1: *z* = 0.134, *P* = 0.893; reader 2: *z* = 0.094, *P* = 0.925).

**Table 5:** The comparison of areas under the curve (AUCs) based on the f-apparent diffusion coefficient (ADC), z-ADC, and s-ADC sets for both readers.

		z-ADC *vs*. s-ADC_b1000_	z-ADC *vs*. f-ADC	s-ADC _b1000_ *vs*. f-ADC
Reader-1	AUC	0.96 *vs*. 0.95	0.96 *vs*. 0.84	0.95 *vs*. 0.84
	*z* value	0.134	2.445	2.207
	*P*-value	0.893	0.015	0.027
Reader-2	AUC	0.94 *vs*. 0.94	0.94 *vs*. 0.80	0.94 *vs*. 0.80
	*z* value	0.094	2.652	2.29
	*P*-value	0.925	0.008	0.022

f-ADC, ADC map derived from full field-of-view (FOV) diffusion-weighted imaging (f-DWI) and all the available b-values (b = 50, 1,000, and 1,500 s/mm^2^); z-ADC, ADC map derived from zoomed FOV diffusion-weighted imaging and all the available b-values (b = 50, 1,000, and 1,500 s/mm^2^); s-ADC_b1000_, ADC map synthesized using our proposed deep learning framework with input from f-DWI (b = 1,000 s/mm^2^).

## Discussion

The main contribution of our study to the literature is the proposed GAN-based ADC synthesis method that can be used to generate s-ADC maps using single *b*-value DWIs with better image quality and stronger ADC calculation accuracy and reproducibility than a full FOV ADC, but without dependence on software, hardware, and additional scanning time that zoomed FOV ADC technology requires. A shorter scan time will lead to better patient comfort and fewer motion artifacts due to involuntary or autonomous motions. The high reproducibility and accuracy of the ADC calculations may effectively reduce the risk of delayed treatment or unnecessary overtreatment due to the misdiagnoses of benign and malignant lesions. Therefore, the GAN-based ADC synthesis method can increase the clinical benefits to patients, reduce treatment times, and lower the costs incurred by patients and hospitals.

In previous studies (31–33), GANs have been used successfully for image-to-image transformations, such as in generating MRI or PET images using CT images or synthesizing CT images from MRI images, and they have performed well in terms of the traditional pixel-wise metrics. However, GAN-generated images do not have a physical meaning, and they can often lead to spurious images (21). As a result, it is difficult for GANs and their extensions to win the trust of clinicians. Therefore, we not only compared traditional pixel-wise metrics, including the RMSE, SSIM, FSIM, and PSNR scores, between the s-ADC sets and reference images, but we also compared the s-ADC and ADC values generated by traditional methods to evaluate the clinical value of GAN-generated images.

In the present study, we evaluated s-ADC maps that were based on DWI inputs with different *b*-values and found that the choice of *b*-values influenced the s-ADC values. Based on a subjective visual evaluation, the s-ADC_b1000_ maps delineated normal tissues and lesions more clearly than the s-ADC_b50_ maps, and they provided more details for targeted images than the s-ADC_b1500_ set. The quantitative evaluation results are also consistent with the visual evaluation results. Among the three s-ADC sets, the s-ADC_b1000_ set achieved a lower RMSE score and higher SSIM, FSIM, and PSNR scores than the s-ADC_b50_ and s-ADC_b1500_ sets, indicating that the s-ADC_b1000_ set is more similar to the realistic z-ADC in terms of noise distribution, image structure, and features. Additionally, the s-ADC_b1000_ set showed better intra-reader repeatability and inter-reader consistency than the s-ADC_b50_ and s-ADC_b1500_ sets. Moreover, the s-ADC_b1000_ set showed the best ADC value inter-method consistency with the z-ADC set, suggesting that a DWI with a *b*-value of 1,000 s/mm^2^ might be more suitable for synthesizing ADC maps than one with a *b*-value of 50 or 1,500 s/mm^2^. The similarity between the target image z-DWI and s-DWI strongly depends on how much useful information the input f-DWI can provide to the generator for the extraction of meaningful features to begin the mapping between f-DWI and z-ADC. Low-*b*-value DWIs suffer from T2 shine-through or black-through effects, whereas high-*b*-value DWIs might be affected by diffusion kurtosis effects (34). These effects have a negative influence on image quality and lesion information, causing a relatively lower similarity between the s-ADC_b50_ and s-ADC_b1500_ sets and the z-ADC set compared to the s-ADC_b1000_ set (3).

In our study, both the z-ADC and s-ADC sets showed less distortion and better reproducibility of the quantitative ADC values for all the evaluated tissues; they also showed better tumor detection and classification capacity than the f-ADC sets. The ADC values are generated for most of the current clinical implementations by calculating the signal intensity decay using two or more DWI sets with different *b*-values (1–5, 9–11, 13, 14). The reproducibility and accuracy of the calculated ADC values are affected by the choice of *b*-values (3, 4, 34) and the DWI image quality (14). The application of a significant number of *b*-values improves the reproducibility and accuracy of the calculated ADC values, although it also increases the scanning time (3, 35). In contrast to traditional ADC calculation methods, our proposed method takes advantage of the ability of GAN to simulate data distribution and synthesize ADC maps that are highly similar to real zoomed FOV ADC maps that use a full FOV DWI with a single *b*-value. Considering the excellent image quality consistency and similar tumor detection and classification abilities between the s-ADC and z-ADC maps, we believe that the deep learning algorithm might be a feasible method for generating ADC maps as an alternative to z-ADC maps without requiring a strong dependence on software, hardware, and additional scan time (36).

Our study has several limitations. First, the s-ADC_b1000_ set showed the best image quality among the s-ADC sets; however, it remains unknown whether a DWI set with a *b*-value of 1,000 s/mm^2^ is the most appropriate for ADC map synthesis. In future studies, s-ADC sets generated using DWI sets with more potential *b*-values should be compared. Second, as ADC values vary across vendors, the generalizability of our model across MRI scanners from different vendors requires multi-center verification.

In conclusion, the GAN-based ADC synthesis method can generate s-ADC maps using a single *b*-value DWI with good image quality and high reproducibility and ADC calculation accuracy.

## Data Availability Statement

The original contributions presented in the study are included in the article/supplementary material. Further inquiries can be directed to the corresponding author.

## Ethics Statement

The studies involving human participants were reviewed and approved by The ethics committee of Shanghai Jiao Tong University Affiliated Sixth People’s Hospital. The patients/participants provided their written informed consent to participate in this study. Written informed consent was obtained from the individual(s) for the publication of any potentially identifiable images or data included in this article.

## Author Contributions

JZ is the guarantor of the integrity of the entire study (study concepts/study design, data acquisition, and data analysis/interpretation). LH participated in statistical analysis, manuscript drafting and manuscript revision for important intellectual content. LH and JZ edited the manuscript. LH and DZ participated in literature research. LH, DZ, YX, and LW participated in clinical studies. All authors contributed to the article and approved the submitted version.

## Funding

This study was funded by the National Natural Science Foundation of China (nos. 81901845 and 81671791), the Science Foundation of Shanghai Jiao Tong University Affiliated Sixth People’s Hospital (no. 201818), and the Shanghai Key Discipline of Medical Imaging (no. 2017ZZ02005).

## Conflict of Interest

CF was employed by Siemens Shenzhen Magnetic Resonance, Ltd. TB was employed by MR Application Predevelopment, Siemens Healthcare GmbH, Erlangen, Germany.

The remaining authors declare that the research was conducted in the absence of any commercial or financial relationships that could be construed as a potential conflict of interest.

## Publisher’s Note

All claims expressed in this article are solely those of the authors and do not necessarily represent those of their affiliated organizations, or those of the publisher, the editors and the reviewers. Any product that may be evaluated in this article, or claim that may be made by its manufacturer, is not guaranteed or endorsed by the publisher.
